# REIMAGINING THE FUTURE OF ASSISTED LIVING TO PROMOTE QUALITY

**DOI:** 10.1093/geroni/igac059.1114

**Published:** 2022-12-20

**Authors:** Sheryl Zimmerman

## Abstract

Assisted living (AL) has existed in the U.S. for decades, evolving in response to older adults’ need for supportive care and distaste for nursing homes and older models of congregate care. Today, AL is the largest provider of residential long-term care in the country, with more than 996,000 AL beds in almost 29,000 AL communities. Unfortunately, that growth has spawned notable concern, with a recent report concluding that the initial key constructs of AL have become mired under key tensions such that “the current model of AL has been taken as far as it can go.” This symposium includes five presentations that respond to some of those tensions. The first two presentations respond to the increased acuity of AL residents: one addresses challenges and recommendations for residents’ palliative care needs based on numerous studies, and the other provides expert consensus panel recommendations for pragmatic medical and mental health care provision in AL. The next two presentations respond to concerns related to quality in AL, one focusing on frameworks, measures, and implementation of quality metrics, the other envisioning use of administrative data to study and promote quality. The final presentation speaks to the complexity of regulation, and how related research can be improved to better examine consequential differences between models of AL care. The discussant, a nationally recognized and highly regarded leader from the American Health Care Association/National Center for AL, will discuss how the presentations can be used to reimagine the future of AL to promote quality.

